# Background data on solar heat-assisted stripping-absorption system for ammonia recovery from food waste digestate

**DOI:** 10.1016/j.dib.2020.106619

**Published:** 2020-12-07

**Authors:** Lucas A.O. Melgaço, Erik Meers, César R. Mota

**Affiliations:** a Department of Sanitary and Environmental Engineering, Federal University of Minas Gerais, Belo Horizonte, Brazil; b Department of Green Chemistry and Technology, Ghent University, Ghent, Belgium

**Keywords:** Nitrogen recovery, Food waste, Solar heat, Stripping-absorption, Anaerobic digestate

## Abstract

The data presented in this paper are related to the research article “Ammonia recovery from food waste digestate using solar heat-assisted stripping-absorption” [1]. The raw and filtered data are associated to daily monitoring of NH_4_ concentration of food waste digestate, pH of digestate and absorption solution and temperature of food waste digestate throughout experiments at different conditions. In addition, data of temperature monitoring in different points of solar-heat assisted stripping-absorption device are presented. The data could help further studies aiming to improve this system. The detailed data of these experiments could help to improve the performance and to reduce costs of nitrogen recovery from digestate using stripping-absorption technology.

## Specifications Table

**Table:** 

Subject	Waste Management and Disposal
Specific subject area	Resource recovery from waste
Type of data	Table
How data were acquired	Ammonium concentration was measured by colorimetric method at 420 nm on UV–Vis Spectrophotometer device. pH were measured using a digital pHmeter Temperature was measured using digital thermocouple thermometer.
Data format	Raw and Filtered data
Parameters for data collection	The data were collected throughout experiments with the solar-heat assisted stripping-absorption device treating food waste digestate.
Description of data collection	Initial and daily ammonium concentration and pHs of treated food waste digestate and absorption solution (0,5 mol/L H_2_SO_4_) were measured at outlet of columns The temperature of food waste digestate was measured daily, only for 45 °C experiments, at 12 pm on different points of the solar-heat assisted stripping-absorption device: (i) inside vacuum glass solar collectors, (ii) inlet stripping column and (iii) outlet stripping column.
Data source location	Department of Sanitary and Environmental Engineering, Federal University of Minas Gerais, Belo Horizonte, Brazil
Data accessibility	With the article
Related research article	L.A.O Melgaço, E. Meers, C.R. Mota, Ammonia recovery from food waste digestate using solar heat-assisted stripping-absorption, Waste Management 113 (2020) 244–250. https://doi.org/10.1016/j.wasman.2020.05.047

## Value of the Data

• This data could help to select different process parameters to improve the performance of nitrogen recovery from biogas digestate based on stripping-absorption technologies.
• This data can help to select best resource recovery technologies to manage biogas digestate and other wastes.
• This data can be used for further studies aiming to optimize and reduce costs for nitrogen recovery by stripping-absorption process.
• This data could help to improve and foster a widespread application of stripping-absorption process for ammonia recovery, mainly aiming nitrogen fertilizer recycling.

## 1 Data Description

The data presented in this article are associated with the article (L.A.O. Melgaço et al., [1]) and were acquired using a solar-heat assisted stripping-absorption device treating food waste digestate (Table 1). The daily monitoring of food waste digestate and absorption solution pH and NH_4_ concentration at different experimental conditions (25 °C and 45 °C – G/L ratios 1700 and 2600 are presented, respectively, in Table 2 and 3. The temperatures at different experimental conditions are presented in Table 4.

**Table 1:** Background information on solar-heat assisted stripping-absorption device.

	Stripping column^a^^,^^b^	Absorption column^a^^,^^c^	Solar-heater
Height (m)	1,80	1,20	–
Diameter (m)	0,15	0,15	–
Total volume (L)	30	17	33

a Stripping and absorption columns were made with PVC tubes.

b stripping column had a packed bad of 1,20 m height (polyethylene raschig rings - diameter: 1.5 cm; length: 5 cm; specific surface area: 300 m^2^.*m* ^−^ ^3^ as packing material).

c absorption column did not have packing material;.

**Table 2:** The daily monitoring of pH of the food waste digestate and absorption solution.

	25 °C – G/L 1700		45 °C – G/L 1700		25 °C – G/L 2600		45 °C – G/L 2600	
	pH		pH		pH		pH	
Time (hours)	Stripping	Absorption	Stripping	Absorption	Stripping	Absorption	Stripping	Absorption
0	8.44	0,88	8,61	0,99	8,15	0,6	8,01	0,6
24	8,57	0,8	8,93	1,09	8,72	0,78*	8,74	0,92
48	8,74	0,87	8,72	1,21	9,11	0,66	8,74	1,01
72	8,71	1,11	8,88	1,42	8,66	0,72	8,78	1,36
96	8,99	1,28	8,97	1,61	9,09	0,84	8,85	1,32
120	9,01	1,25	9,11	1,81	9,06	0,82	8,7	1,45
144	9,04	1,17	9,19	2,03	9,07	0,79	8,49	1,56
168	9,07	1,12	9,15	2,14	9,03	0,77	8,61	1,62

⁎ replaced absorption solution.

**Table 3:** The daily monitoring of NH_4_ concentration of food waste digestate at different experimental conditions.

	25 °C – G/L 1700	45 °C – G/L 1700	25 °C – G/L 2600	45 °C – G/L 2600
Time (hours)	NH4-N (mg/L)			
0	1302,10	1241,77	1385,61	1275,58
24	727,43	746,43	1139,90	886,77
48	461,77	458,10	688,47	722,77
72	144,43	360,77	313,71	557,10
96	102,77	263,43	77,10	381,77
120	95,30	166,10	65,13	353,10
144	83,77	104,43	44,76	203,10
168	74,77	18,43	31,78	53,49

**Table 4:** The temperatures at different experimental conditions.

	Temperature (°C)^a^			Temperature (°C)^b^		
	Sampling point^c^			Sampling point		
Time (hours)	I	II	III	I	II	III
0	60	43	30	63	47	31
24	65	48	33	62	46	33
48	63	45	32	63	44	29
72	66	42	28	65	45	34
96	62	47	34	61	43	30
120	65	45	32	60	45	29
144	64	44	28	64	42	32
168	65	41	30	62	44	30

a experimental conditions: 45 °C and G/L 1700.

b experimental conditions: 45 °C and G/L 2600.

c sampling point are: (I) inside vacuum glass solar collectors, (II) inlet stripping column and (III) outlet stripping column.

## 2 Experimental Design, Materials and Methods

The system was operated at an open-loop, semi-batch mode with different operational conditions (temperature 25 °C and 45 °C; gas/liquid ratios 1700 and 2600). The liquid digestate (LD) was carried to the top of the stripping column by a dosing pump, and a plastic shower inside the column was used as an LD distribution device. The LD percolated through the packed bed inside stripping column and was collected in a reservoir at the bottom of the column for recirculation. Air was injected in the stripping column at countercurrent flow (340 L/min), and the changes in G/L ratios were achieved by decreasing LD flow rate. The top of the column was closed to prevent the release of gaseous effluent into the atmosphere. A plastic tube connected the top of the stripping column with the absorption column, where the NH_3_-rich air entered from the bottom and left from the top. Sulfuric acid solution (0.18 mol/L) was continually recycled in countercurrent flow of 0.1 L/min using a dosing pump to recover the striped ammonia as (NH_4_)_2_SO_4_. All batch runs lasted 7 days and were carried out in duplicates.

For the tests that were performed at 45 °C, a solar heater device (vacuum glass solar collectors) was coupled to the air-stripping column, replacing the liquid digestate reservoir. Liquid digestate that came out of the stripping column flowed by gravity to the solar heater and was recycled back to the top of the stripping column using a dosing pump.

Samples from the liquid fraction of digestate and sulfuric acid solution were taken daily from the bottom of each column and analyzed for pH and ammonia-nitrogen (NH_4_—N) content. The pH was measured using a digital pHmeter (DIGIMED model DM-44) and ammonia-nitrogen analysis was made based on colorimetric phenol method described in *Standard Methods for Examination of Water and Wastewater* (APHA et al., 2012) [2].

The temperature were measured daily at 12pm using a portable thermocouple digital thermometer. The measuring were made in different points of the solar-heat assisted stripping-absorption device: (i) inside vacuum glass solar collectors, (ii) inlet stripping column and (iii) outlet stripping column.

## CRediT Author Statement

**Lucas A.O. Melgaço** Conceptualization, Methodology, Investigation, Formal Analysis, Writing–Original Draft; **Erik Meers:** Methodology, Writing–Review and Editing, Supervision, Funding acquisition; **César R. Mota:** Writing–Review and Editing, Supervision.

## Declaration of Competing Interest

The authors declare that they have no known competing financial interests or personal relationships, which have, or could be perceived to have, influenced the work reported in this article.

## Appendix Supplementary materials

Image, application 1
